# Effects of glycyrrhetinic acid on production performance, serum biochemical indexes, ruminal parameters, and rumen microflora of beef cattle

**DOI:** 10.3389/fvets.2025.1529383

**Published:** 2025-03-26

**Authors:** Long Wang, Mingren Qu, Lin Li, Wenliang Mei, Fengwei Zhang, Ziyu Hu, Geping Li, Lanjiao Xu, Huan Liang

**Affiliations:** ^1^Jiangxi Key Laboratory of Animal Nutrition, Nanchang, Jiangxi, China; ^2^Shenglong Cattle Industry Group Co., Ltd, Pingxiang Jiangxi, China

**Keywords:** beef cattle, glycyrrhetinic acid, growth performance, microbiome, rumen fermentation

## Abstract

This research was carried out to assess the impact of supplementing with glycyrrhetinic acid (GA) on production performance, serum biochemical indexes, ruminal parameters, and rumen bacterial flora of beef cattle. Twenty-four Simmental bulls were randomly assigned to two dietary treatments (*n* = 12 per treatment): the control treatment (basal ration, CON) and the GA treatment (basal ration supplemented with GA at 0.1% DM). After an 87-day feeding trial (7-day adaptation period and 80-day period dedicated to data and sample collection), feces, blood, and rumen fluid samples were collected on day 87. The GA addition significantly increased the average daily gain of beef cattle (*p* < 0.05). The GA treatment exhibited significantly greater apparent digestibility of crude protein, neutral detergent fiber, and acid detergent fiber than the control treatment (*p* < 0.05). Total volatile fatty acid concentration, microbial protein concentration, and propionic acid concentration in the rumen fluid were significantly increased by GA addition (*p* < 0.05). Compared with the control group, the interleukin-4 concentration was significantly higher in GA treatment (*p* < 0.05). The indices, including operational taxonomic units (OTUs), Sobs, Shannon, Ace, and Chao1, were found to be greater in the GA treatment. At the phyla level, GA addition (*p* < 0.05) significantly decreased the relative abundance of Bacteroidetes and increased the relative abundance of Firmicutes, while also significantly decreasing the Bacteroidetes:Firmicutes ratios. At the genera level, the relative abundance of *Prevotella, NK4A214_group, norank_f_UCG-011, Prevotellaceae_UCG-003, Christensenellaceae_R-7_treatment, Prevotellaceae_UCG-001, norank_f_Bacteroidales_UCG-001, Pseudobutyrivibrio*, and *Butyrivibrio* significantly differed due to GA addition (*p* < 0.05). Carbohydrate and amino acid transport and metabolism, as well as energy production and conversion, were significantly enriched in the GA treatment (*p* < 0.05). In summary, the findings indicated that adding glycyrrhetinic acid to the diet could improve growth performance and modify the rumen microbial composition and diversity of beef cattle.

## 1 Introduction

With the significant reduction of antibiotics to the feed in China, the search for natural alternatives is useful. Natural plant extracts contain a rich variety of active ingredients, such as flavonoids, essential oils, alkaloids, polyphenols, polysaccharides, and saponins. Those active ingredients have the characteristics of low resistance, low toxicity, low residues, and antioxidation. Therefore, as a type of “safe, efficient, and stable” feed additive, natural plant-based feed additives have gradually become the preferred choice for antibiotic replacement in livestock farms (1, 2). Numerous studies have shown that natural plant-based feed additives have the following effects on livestock: promoting growth, improving animal production performance, enhancing immunity, enhancing stress resistance, improving intestinal health, and improving the quality of animal products. Some plants have been proven to have good medicinal value (3). Hence, the exploration of feed additives derived from plants holds considerable importance for the sustainable growth of the livestock sector as well as for the enhancement and improvement of the quality of animal products.

Glycyrrhiza (*Glycyrrhiza uralensis* Fisch.) is a perennial herb belonging to the legume family, characterized by its distinctively sweet-tasting rhizomes. It originated in the Mediterranean and is now widely distributed in Europe, Asia, Australia, the United States, and other places. Glycyrrhiza is a Chinese herbal medicine widely used in Chinese medicine and livestock production that has extremely rich biological functions. The primary bioactive constituents found in glycyrrhiza, include triterpenes, flavonoids, and polysaccharides (4, 5). As per the quality index of glycyrrhiza, glycyrrhetinic acid (GA) is an active triterpenoid saponin isolated from glycyrrhiza and is the compound with the majority content in glycyrrhiza. Research indicates that GA exhibits anti-inflammatory (6, 7), antibacterial (8, 9), immunomodulatory, and other pharmacological properties (10, 11).

In recent years, GA has increasingly been utilized as supplementary feed in the production of livestock. Tian (12) indicated that incorporating GA into the total mixed ration (TMR) diet of cows during the perinatal period reduced the levels of serum interleukin 12, interleukin 1, and interleukin 6, increased the levels of interleukin 2, and increased the levels of serum total antioxidant capacity, as well as superoxide dismutase, glutathione peroxidase, and catalase oxidase activity. GA enhances the body’s anti-inflammatory and antioxidant capacity and improves production performance. Jiang (13) indicated that incorporating GA into the diet notably enhanced feed utilization and production performance, elevated propionic acid levels, and significantly decreased the concentrations of NH_3_-N, acetic acid, and the acetic to propionic acid ratio in the rumen fluid of Karakul sheep. The addition of GA also reduced the abundance of rumen microorganisms, which are related to methane emission (13). Other studies have found that GA could improve the feed-to-meat ratio, immunity, and performance in livestock production (14, 15). Consequently, it can be concluded that GA possesses the potential to serve as a beneficial feed additive aimed at enhancing both the intestinal health and overall performance of beef cattle.

However, there are only a few studies reported about the effect of glycyrrhetinic acid on beef cattle. Therefore, in this experiment, glycyrrhetinic acid was added to beef cattle feed to determine its effects on growth performance, apparent nutrient digestibility, blood biochemical indicators, rumen fermentation parameters, and rumen microflora to provide a theoretical basis for the application of glycyrrhetinic acid in beef cattle production.

## 2 Materials and methods

### 2.1 Animals, diets, and experimental design

Cattle selected for this experiment were provided by a commercial beef cattle farm (Jiangxi Agriculture University Test Base, Xinyu, China). The glycyrrhetinic acid was provided by Beijing Centre Technology (Beijing, China). Twenty-four Simmental cattle (initial mean ± SE: 588 ± 54.6 kg of body weight, and 15 ± 1 month old) were randomly divided into two treatments (*n* = 12 per treatment): control treatment and GA treatment. All the cattle were fed individually, and the control treatment cattle were fed the standard diet, while those in the GA treatment group were fed the standard diet +0.1% glycyrrhetinic acid (the dosage of glycyrrhetinic acid added was determined from previous rumen *in vitro* fermentation experiments). Cattle received the feed two times a day at 8:00 a.m. and 4:00 p.m., and clean, fresh water was provided as required. All treatments lasted 80 days, and the first 7 days were allocated for adaptation. The specific formulation and nutritional components of the standard diet utilized throughout the study can be found in Table 1.

**Table 1:** Components and nutritional composition of the basal diet.

Ingredients/Nutrient level	%
Corn	38.88
Soybean meal	6.80
Cottonseed meal	4.80
Rapeseed meal	3.20
Sunflower meal	1.52
DDGS^1^	3.60
Limestone	0.20
NaHCO_3_	0.70
NaCl	0.30
Premix^2^	1.00
Wheat straw	40.00
Total	100.00
NE_mf_^3^/(MJ/kg)	6.85
Crude protein	13.50
Neutral detergent fiber	37.89
Acid detergent fiber	20.95
Ca	1.02
P	0.62

^1^DDGS, distillers dried grains with solubles. ^2^The pre-mix provided the following nutrients per kg of diet: VA 30000000 IU, VD3 1000000 IU, VE 80000 IU, VK3 10 g, VB1 10 g, VB2 25 g, Cu 0.04 g, Fe 0.17 g, Zn 0.088 g, Mn 0.064 g, Co 0.02 g, I 0.03 g. ^3^NEmf was calculated according to the Chinese Beef Cattle Feeding Standard (NY/T815-2004), while the other nutrient levels were measured values.

### 2.2 Growth performance and serum biochemical parameters

At both the commencement and conclusion of the experiment, each animal was weighed individually in the morning before they were given their feed. To determine the average daily gain (ADG), the total weight gain for each individual cattle was divided by the number of days the trial lasted. Additionally, the average daily dry matter intake (ADMI) was assessed by taking the total amount of diet provided to each cattle, subtracting the leftover feed, and then dividing this figure by the total duration of the trial in days. The residue was collected and weighed before morning feeding. The feed conversion rate was ADMI divided by ADG. On the concluding day of the experiment, blood samples were collected from the caudal vein of each cattle just before their morning feeding. After standing for 30 min, the blood samples underwent centrifugation at a speed of 3,500 revolutions per min for 15 min. Following this, the serum was isolated and preserved at −20°C for subsequent analysis. The levels of serum total protein (TP), albumin (ALB), globulin (GLB), total cholesterol (TC), triglycerides (TG), high-density lipoprotein (HDL), low-density lipoprotein (LDL), alanine aminotransferase (ALT), aspartate aminotransferase (AST), lactate dehydrogenase (LDH), and alkaline phosphatase (ALP), along with interleukin-4 (IL4) and interleukin-6 (IL6), were detected by automatic biochemical analyzer (3,100 Hitachi, China).

### 2.3 Rumen fluid sample collection and measurements

The diet offered and refused was measured and sampled daily. Between 78 and 80 days, the total amount of feces excreted by each cattle was recorded, and a fecal sample (1/10 of the wet weight) was collected and mixed with sulfuric acid solution (100 mL/L). TMR, refusals, and fecal samples were subjected to drying in an oven set at a temperature of 65°C for a duration of 48 h. After the drying process, the samples were carefully ground to achieve a uniform consistency that enabled them to pass through a 1-mm sieve for further analysis.

Before feeding on the 80th day, rumen fluid was collected (150 mL) utilizing an oral stomach tube, following the protocol established by Shen et al. (16). Following collection, the rumen fluid was immediately flash-frozen in liquid nitrogen to preserve its integrity and biochemical composition. The frozen samples were then securely stored at a temperature of −80°C for later analysis. In addition to the preservation of the samples, a portion of the rumen fluid was utilized to measure volatile fatty acids (VFAs), using the technique described by Hu et al. (17). In summary, ortho-phosphoric acid (25% w/v) was introduced to the rumen fluid sample prior to filtration and centrifugation (12,000 *g* for 10 min at 4°C), after which VFAs were examined using gas chromatography (GC-2014 Shimadzu, Japan).

### 2.4 Nutrient apparent digestibility

The analysis of diet, refusals, and fecal samples focused on several parameters, including acid-insoluble ash (AIA), dry matter (DM), crude protein (CP), neutral detergent fiber (NDF), acid detergent fiber (ADF), and ether extract (EE). The AIA served a crucial role as an internal marker, facilitating the estimation of total fecal output, which is essential for determining apparent nutrient digestibility coefficients. To ensure the reliability of the AIA measurement, the procedure was conducted using the 2 N HCl method in triplicate, thereby enhancing the validity of the results obtained (18). In addition to the analyses involving AIA, all the remaining sample examinations were performed in duplicate to ensure the accuracy and reproducibility of data. The laboratory dry matter content was determined by subjecting the samples to a drying process at 105°C in a forced-air oven for a duration of 24 h. The ash content was evaluated to determine organic matter (OM) by incinerating the samples at 550°C for a period of 4 h (19). Neutral detergent fiber and acid detergent fiber were detected using the Van Soest method by the ANKOM^200^ fiber analyzer (A200i Ankom, United States), with the addition of sodium sulfite and *α*-amylase for the NDF procedure. Crude protein was determined using the Kjeldahl method by the FOSS Kjeltec 8,400 Nitrogen Analyzer (8,400 FOSS, Denmark). The ether extract was evaluated using Soxhlet extraction (20). Apparent total tract digestibility CP, NDF, ADF, and EE were determined from the following equation: 100–100 × [(C_AIA-feed_/C_AIA-feces_) × (C_nutrient-feces_/C_nutrient-feed_)], where C_AIA-feed_ represents the concentration of AIA in the feed, C_AIA-feces_ represents the concentration of AIA in the feces, C_nutrient-feces_ represents the concentration of nutrient in the feces, and C_nutrient-feed_ represents the concentration of nutrient in the feed.

### 2.5 DNA extraction and sequencing

E.N.Z.A Stool DNA Kit method were used to extract total DNA from microbial sources in rumen fluid. The isolation process was carried out in strict adherence to the instructions provided by the manufacturer. The quantity and purity of isolated DNA were measured on an ND-1000 spectrophotometer (NanoDrop, United States). The amplification of the eukaryotic ribosomal RNA gene’s 16S rDNA V3–V4 region was achieved using polymerase chain reaction (PCR), incorporating an initial step at 95°C for 3 min, followed by 30 cycles consisting of 30 s at 95°C,30 s at 55°C, and 45 s at 72°C, culminating with a final extension at 72°C for 10 min (eventually stopped by the user). Specific primers were used in this process, namely 338F (5’-ACTCCTACGGGAGGCAGCAG-3′) and 806R (5’-GGACTACHVGGGTWTCTAAT-3′). The PCR reactions were executed in triplicate to ensure the reliability and reproducibility of the results. Each 20 μL of the reaction mixture was carefully prepared, incorporating 4 μL of 5× FastPfu buffer, 2 μL of 2.5 mM dNTPs, and equal volumes of both the forward and reverse primers, each at a concentration of 5 μM. Additionally, the mixture contained 0.4 μL of FastPfu polymerase, 0.2 μL of BSA, and 10 ng of template DNA, which served as the starting material for the amplification process.

PCR product purification used an AxyPrep DNA Gel Extraction Kit (Axygen, United States), following the guidelines provided by the manufacturer. Additionally, quantification was performed with a Quantus™ Fluorometer (Promega, United States). Purified amplicons were pooled in equimolar and paired-end sequenced on an Illumina NovaSeq PE250 platform (Illumina, United States) according to the standard protocols by Majorbio Bio-Pharm Technology Co. Ltd. (Shanghai, China). The initial sequence reads were submitted to the NCBI database (Accession Number: PRJNA884686).

### 2.6 Processing of sequencing data

The raw sequencing reads of the 16S rRNA gene underwent demultiplexing, and the quality of the sequences was assessed and filtered using fastp version 0.20.0 (21), and the filtered reads were merged using FLASH version 1.2.7 (22). Operational taxonomic units (OTUs) were grouped based on a similarity threshold of 97% (23, 24) utilizing UPARSE version 7.1 (23), with the identification and removal of chimeric sequences. Each representative sequence of the OTUs was classified taxonomically through RDP Classifier version 2.2 (25), referencing the 16S rRNA database (e.g., Silva v138) and applying a confidence threshold of 0.7.

### 2.7 Statistical analysis

The analysis of the experimental data was conducted using SPSS 26.0 (SPSS, United States), where independent sample t-tests were used to assess the significance of growth performance, nutrient apparent digestibility, and serum biochemical parameters. The final results are presented as mean values, with differences regarded as showing a tendency when 0.05 < *p* < 0.10 and considered statistically significant at a *p*-value of ≤0.05.

## 3 Results

### 3.1 Growth performance

Table 2 illustrates the impact of GA on the growth performance of beef cattle. When compared to the control (Con) treatment, the inclusion of GA significantly enhanced the average daily gain (*p* < 0.05). The average daily dry matter intake in GA treatment indicates a potential to be greater than in the Con treatment (*p* < 0.10).

**Table 2:** Effects of GA on the growth performance of beef cattle.

Item^1^	Treatment		SEM^2^	*p*-value
	Con	GA		
IBW, kg	282	285	21.62	0.946
FBW, kg	363	383	19.34	0.549
ADG, kg/day	1.08	1.30	0.12	0.044
ADMI, kg/day	8.00	8.60	0.31	0.076
FCR	7.69	6.74	0.66	0.174

^1IBW, initial body weight; FBW, final body weight; ADG, average daily gain; ADMI, average daily dry matter intake; FCR, feed conversion rate. 2SEM, standard error of the mean.^

### 3.2 Serum biochemical parameters

The effects of GA on the serum biochemical parameters of beef cattle are shown in Table 3. The concentrations of TP, ALB, GLB, TC, TG, HDL, LDL, AST, ALT, LDH, ALP, and UREA in the serum of the Con group were not significantly different from the GA group. The IL4 concentration of the GA group was significantly higher than that in the Con treatment (*p* < 0.05), and the IL6 concentration tended to increase (*p* < 0.10).

**Table 3:** Effects of GA on the serum biochemical parameters of beef cattle.

Item^1^	Treatment		SEM^2^	*P*-value
	Con	GA		
TP, g/L	71.66	70.44	1.86	0.625
ALB, g/L	30.87	30.45	1.01	0.740
GLB, g/L	40.79	39.99	2.20	0.781
TC, mmol/L	2.85	2.69	0.25	0.649
TG, mmol/L	0.25	0.28	0.02	0.383
HDL, mmol/L	1.84	1.73	0.13	0.584
LDL, mmol/L	0.53	0.57	0.08	0.689
AST, U/L	97.05	94.57	9.57	0.823
ALT, U/L	37.46	40.26	2.63	0.362
LDH, U/L	1552.47	1411.90	45.06	0.188
ALP, U/L	201.91	159.52	43.59	0.386
IL4, pg./ml	8.30	8.96	0.13	0.010
IL6, pg./ml	159.27	177.39	5.83	0.057
UREA, mmol/L	4.33	4.64	0.50	0.557

^1TP, total protein; ALB, albumin; GLB, globulin; TC, total cholesterol; TG, triglyceride; HDL, high-density lipoprotein; LDL, low-density lipoprotein; ALT, alanine aminotransferase; AST, aspartate aminotransferase; LDH, lactate dehydrogenase; ALP, alkaline phosphatase; IL4, interleukin 4; IL6, interleukin 6. 2SEM, standard error of the mean.^

### 3.3 Nutrient apparent digestibility

As shown in Table 4, the apparent digestibility of crude protein, neutral detergent fiber, and acid detergent fiber in the feed of the GA treatment was significantly higher when compared to the Con treatment (*p* < 0.05).

**Table 4:** Effects of GA on the apparent nutrient digestibility of beef cattle.

Item	Treatment		SEM^1^	*P*-value
	Con	GA		
Dry matter (%)	73.5	74.10	1.63	0.636
Crude protein (%)	69.91	78.56	1.59	0.001
Ether extract (%)	79.28	80.63	2.20	0.557
Neutral detergent fiber (%)	63.31	71.94	2.92	0.018
Acid detergent fiber (%)	61.00	70.71	3.72	0.031

^1SEM, standard error of the mean.^

### 3.4 Rumen fermentation characteristics

The rumen pH, isovaleric acid portion, and the ratio of acetic acid to propionic acid in the GA treatment were significantly lower than those observed in the Con treatment (*p* < 0.05, Table 5). In contrast to the Con treatment, the addition of GA significantly enhanced the total VFAs, the concentration of microbial proteins, and the content of propionic acid (*p* < 0.05).

**Table 5:** Effects of GA on cattle rumen pH and volatile fatty acids.

Item	Treatment		SEM^2^	*P*-value
	Con	GA		
Rumen pH	6.75	6.04	0.18	0.001
Acetic acid (%)	26.32	25.19	0.26	0.131
Propionic acid (%)	20.85	23.93	0.49	0.009
Isobutyric acid (%)	2.86	2.49	0.13	0.041
Butyric acid (%)	28.10	27.88	1.08	0.183
Isovaleric acid (%)	8.10	6.93	0.30	0.044
Valeric acid (%)	13.77	13.58	0.44	0.384
TVFA^1^ (mM)	81.56	114.59	9.06	0.007
Acetate: propionate	1.26	1.05	0.04	0.017
Microprotein (mg/dL)	51.13	79.46	7.51	0.005
Ammonia nitrogen (mg/dL)	15.01	17.33	1.66	0.200

^1TVFA, total volatile fatty acids. 2SEM, standard error of the mean.^

### 3.5 Rumen bacterial communities

**Figure 1:**
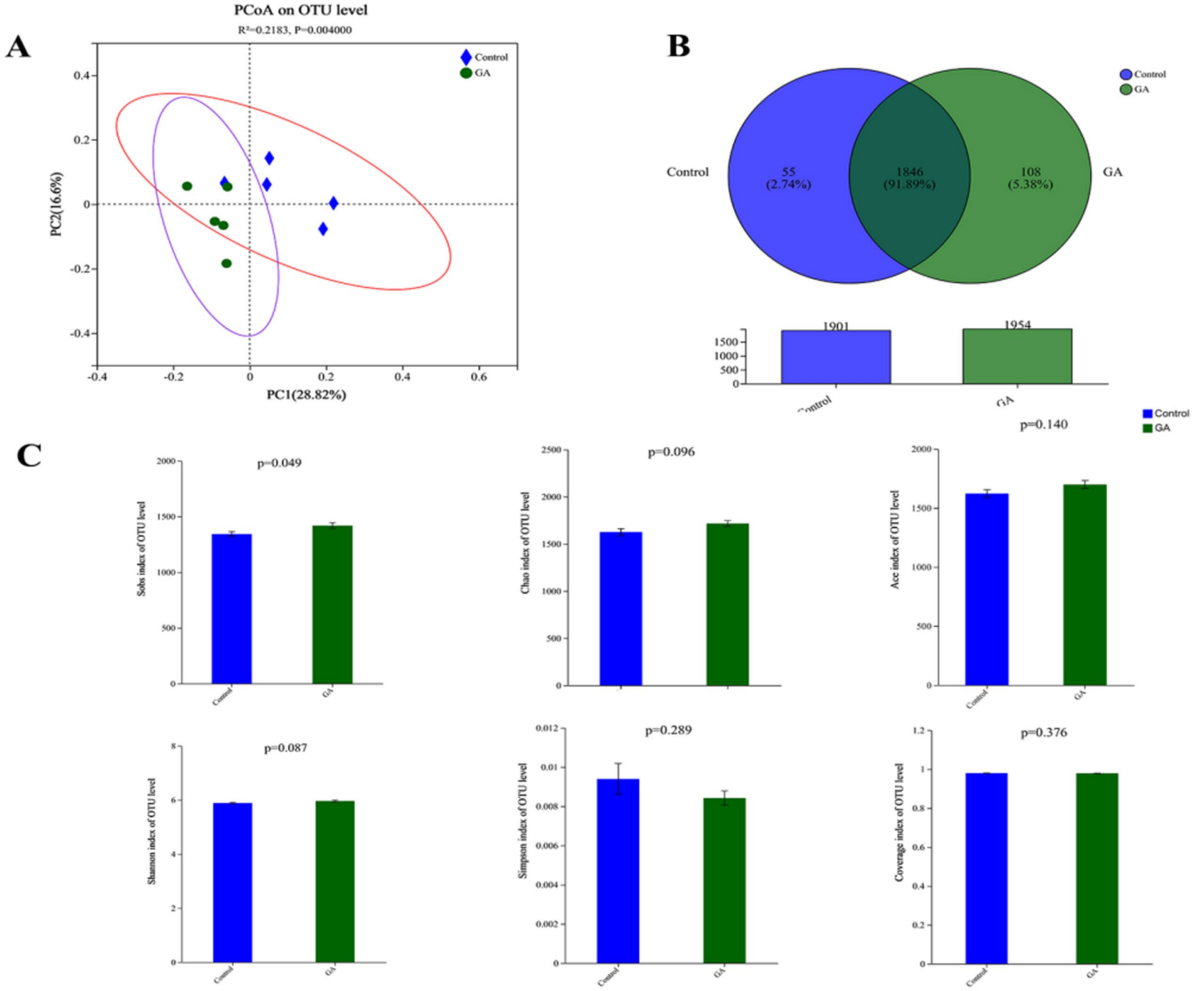
**(A)** Unweighted principal coordinate analysis (PCoA) of taxonomic classifications of rumen bacterial communities in GA and CON treatments. **(B)** Venn diagram of OTUs in GA and CON treatments. **(C)** Alpha-diversity index measures (Student’s *t*-test).

The findings from sequencing analysis are illustrated in Figure 1A. Good coverage for the detected OTUs was 97.92 ± 0.09, and the rarefaction curves displayed distinct asymptotes, suggesting that the community had been nearly completely sampled. Additionally, the unweighted principal coordinates analysis (PCoA) demonstrated a clear separation between the two groups at the OTU level, with statistical significance reported (*p* < 0.01). The similarities in the bacterial community between samples were compared by ANOSIM on Bray–Curtis and revealed significant differences in rumen microbiota structure among different rumen fluid samples (Figure 1B). There were 2009 OTUs identified in GA and control treatments. There were 1954 OTUs and 1901 OTUs in the GA treatment and control treatment, respectively. A total of 1846 OTUs were identified in both treatments, comprising 91.89% of all OTUs. The Sobs index was notably greater in the GA treatment compared to the control treatment (*p* < 0.05), while the Shannon and Chao1 indices showed a trend toward being higher in the GA treatment relative to the control (Shannon *p* < 0.10; Chao1 *p* < 0.10). The GA treatment had a lower Simpson index and a higher Ace index, but neither was significantly different between the two treatments (Figure 1C).

**Figure 2:**
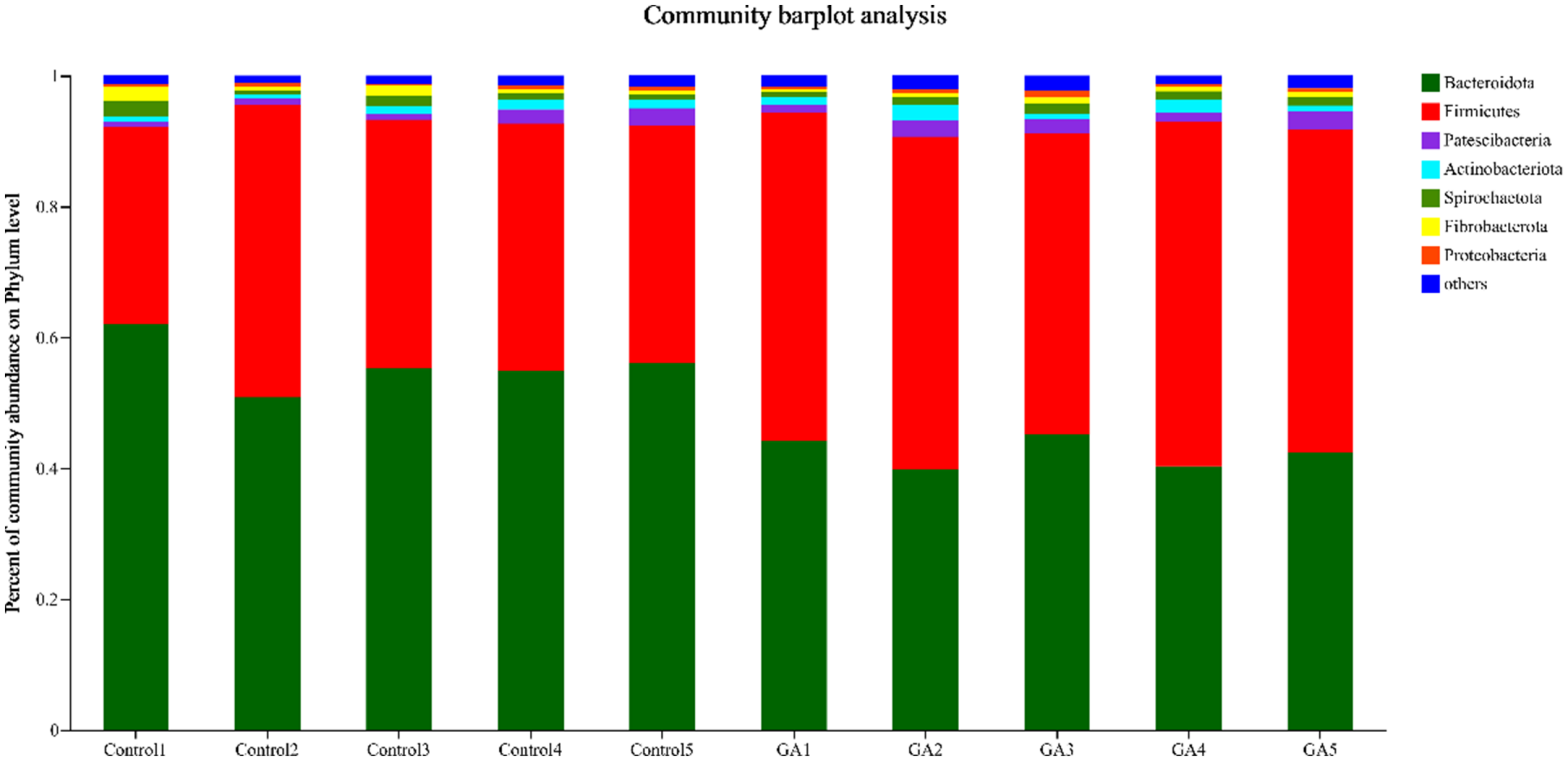
Phylum-level rumen microbiota taxonomic profiling. Different colors represent different phyla of microorganisms under the phylum-level classification, and the area represents the relative abundance of the corresponding microorganism phylum in the sample.

In the analysis of rumen fluid samples, a total of 21 distinct bacterial phyla were identified, and those with relative abundance >1% included *Bacteroidota, Firmicutes, Patescibacteria, Spirochaetota, Actinobacteriota, Fibrobacterota*, and *Proteobacteria* (Figure 2). *Bacteroidota* and *Firmicutes* had the highest relative abundance in both treatments. The GA treatment exhibited a significantly lower relative abundance of *Bacteroidota* compared to the CON group (*p* < 0.01), whereas the relative abundance of *Firmicutes* was significantly greater (*p* < 0.01, Table 6). In the analysis of rumen fluid samples, a total of 21 distinct bacterial phyla were identified. The relative abundances of *Patescibacteria, Actinobacteriota*, and *Proteobacteria* increased by 30.92, 40.74, and 14.00%, respectively, compared with the Con group, but they were not significantly different. Additionally, the *Bacteroidetes* and *Firmicutes* ratio in GA treatment was significantly lower than that in Con treatment (*p* < 0.01).

**Table 6:** Relative abundance of dominant phyla in cattle rumen fluid for different treatments (%).

Item	Treatment		SEM^1^	*P*-value
	Con	GA		
*Bacteroidota*	55.78	42.33	2.08	0.001
*Firmicutes*	37.40	49.81	2.56	0.001
*Patescibacteria*	1.52	1.99	0.48	0.361
*Actinobacteriota*	1.08	1.52	0.35	0.261
*Spirochaetota*	1.29	1.23	0.34	0.882
*Fibrobacterota*	1.06	0.68	0.33	0.305
*Proteobacteria*	0.50	0.57	0.15	0.645
*B/F^2^*	1.53	0.85	0.15	0.002

^1SEM, standard error of the mean. 2B/F, Bacteroidota:Firmicutes ratio.^

**Figure 3:**
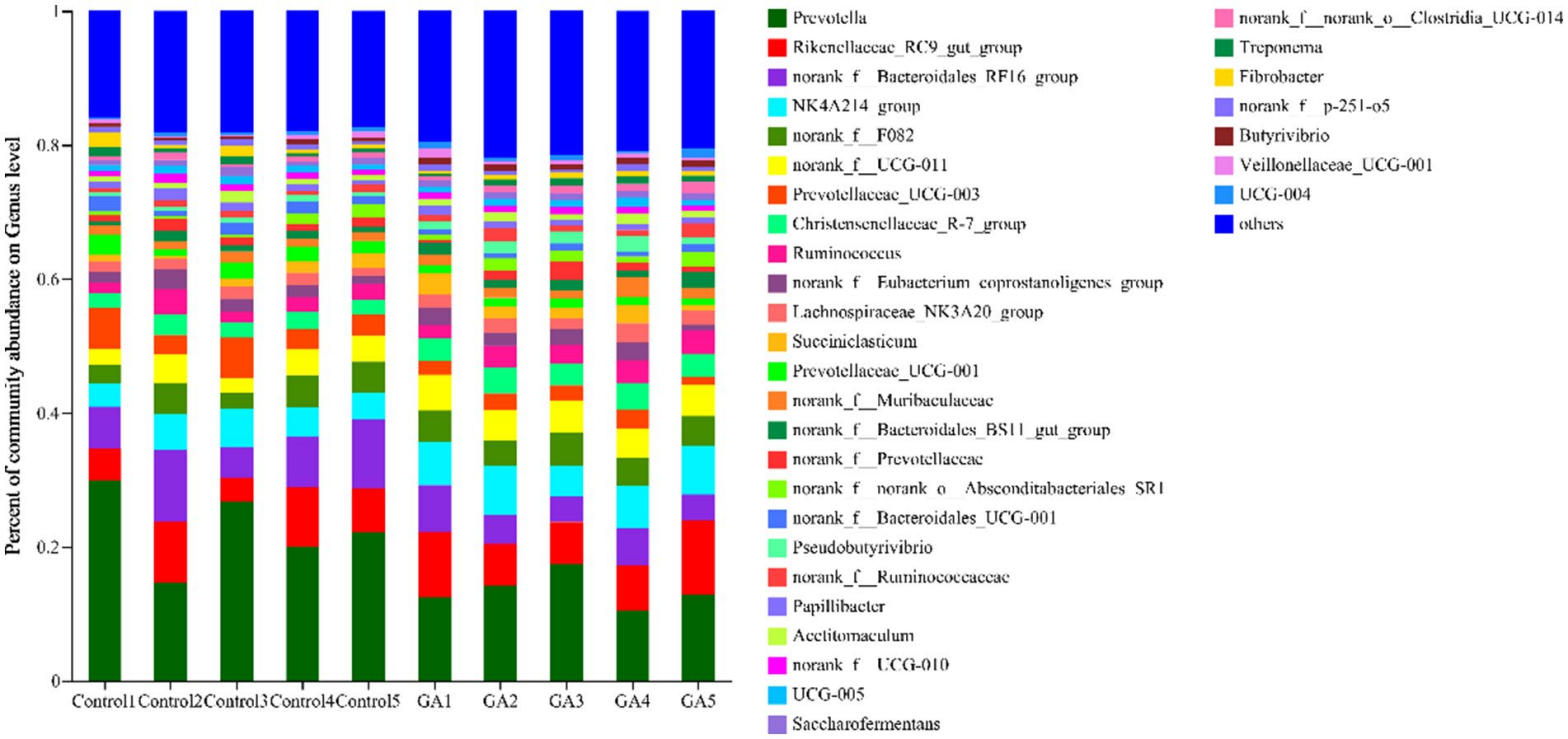
Genus-level rumen microbiota taxonomic profiling. Different colors represent different genera of microorganisms under the genus-level classification, and the area represents the relative abundance of the corresponding microorganism genus in the sample.

A total of 241 bacterial genera were identified, 32 of which had a relative abundance >1% (Figure 3), including *norank_f__Bacteroidales_RF16_group, norank_f__F082, norank_f__Muribaculaceae, norank_f__Bacteroidales_BS11_gut_ group, norank_f__Prevotellaceae, norank_f__Bacteroidales_UCG-001* and *norank_f__p-251-o5* in the *Bacteroidota* phylum*, norank_f__UCG-011, norank_f__Eubacterium_coprostanoligenes_ group, norank_f__Ruminococcaceae, norank_f__UCG-010, UCG-005, norank_f__norank_o__Clostridia_UCG-014* and *UCG-004* in *Firmicutes, norank_f__norank_o__Absconditabacteriales_SR1* in *Patescibacteria, Treponema* in *Spirochaetota,* and *Fibrobacter* in *Fibrobacterota.* The relative abundance of *Prevotella* (*p* < 0.05)*, NK4A214_ group* (*p* < 0.05)*, norank_f__UCG-011* (*p* < 0.05)*, Prevotellaceae_UCG-003* (*p* < 0.05), *Christensenellaceae_R-7_treatment* (*p* < 0.01)*, Prevotellaceae_UCG-001* (*p* < 0.05), *norank_f__Bacteroidales_UCG-001* (*p* < 0.05), *Pseudobutyrivibrio* (*p* < 0.01), and *Butyrivibrio* (*p* < 0.01) significantly differed between the two groups, and the relative abundance of *norank_f__Bacteroidales_RF16_ group* (*p* < 0.10) and *Lachnospiraceae_NK3A20_ group* (*p* < 0.05) tended to differ (Table 7).

**Table 7:** Relative abundance of dominant genera in cattle rumen fluid for different treatments (%).

Phylum	Genus	Treatment		SEM^1^	*p*-value
		CON	GA		
*Bacteroidota*	*Prevotella*	22.62	13.46	2.89	0.013
	*Rikenellaceae_RC9_gut_group*	6.62	8.04	1.48	0.365
	*norank_f__Bacteroidales_RF16_group*	7.87	4.91	1.34	0.057
	*norank_f__F082*	3.85	4.39	0.57	0.384
	*Prevotellaceae_UCG-003*	4.25	2.21	0.081	0.035
	*Prevotellaceae_UCG-001*	2.07	1.18	0.34	0.030
	*norank_f__Muribaculaceae*	1.33	1.78	0.34	0.191
	*norank_f__Bacteroidales_BS11_gut_ group*	0.99	1.56	0.29	0.087
	*norank_f__Prevotellaceae*	1.25	1.29	0.41	0.940
	*norank_f__Bacteroidales_UCG-001*	1.61	0.90	0.26	0.025
	*norank_f__p-251-o5*	0.78	0.75	0.16	0.858
*Firmicutes*	*NK4A214_ group*	4.60	6.38	0.63	0.023
	*norank_f__UCG-011*	3.30	4.73	0.45	0.013
	*Christensenellaceae_R-7_ group*	2.48	3.58	0.23	0.001
	*Ruminococcus*	2.31	2.95	0.47	0.204
	*norank_f__Eubacterium_coprostanoligenes_ group*	1.88	2.11	0.45	0.631
	*Lachnospiraceae_NK3A20_ group*	1.60	2.17	0.25	0.051
	*Succiniclasticum*	1.34	2.01	0.52	0.236
	*Pseudobutyrivibrio*	0.67	1.62	0.24	0.004
	*norank_f__Ruminococcaceae*	0.89	1.32	0.30	0.192
	*Papillibacter*	1.12	1.04	0.20	0.722
	*Acetitomaculum*	0.98	1.14	0.23	0.502
	*norank_f__UCG-010*	1.01	0.97	0.13	0.750
	*UCG-005*	0.96	0.99	0.14	0.853
	*Saccharofermentans*	0.92	1.00	0.12	0.505
	*norank_f__norank_o__Clostridia_UCG-014*	0.75	1.11	0.21	0.131
	*Butyrivibrio*	0.52	0.96	0.06	0.001
	*Veillonellaceae_UCG-001*	0.52	0.71	0.23	0.441
	*UCG-004*	0.47	0.75	0.20	0.197
*Patescibacteria*	*norank_f__norank_o__Absconditabacteriales_SR1*	1.01	1.51	0.44	0.289
*Spirochaetota*	*Treponema*	0.84	0.90	0.25	0.806
*Fibrobacterota*	*Fibrobacter*	1.06	0.67	0.33	0.297

^1SEM, standard error of the mean.^

**Figure 4:**
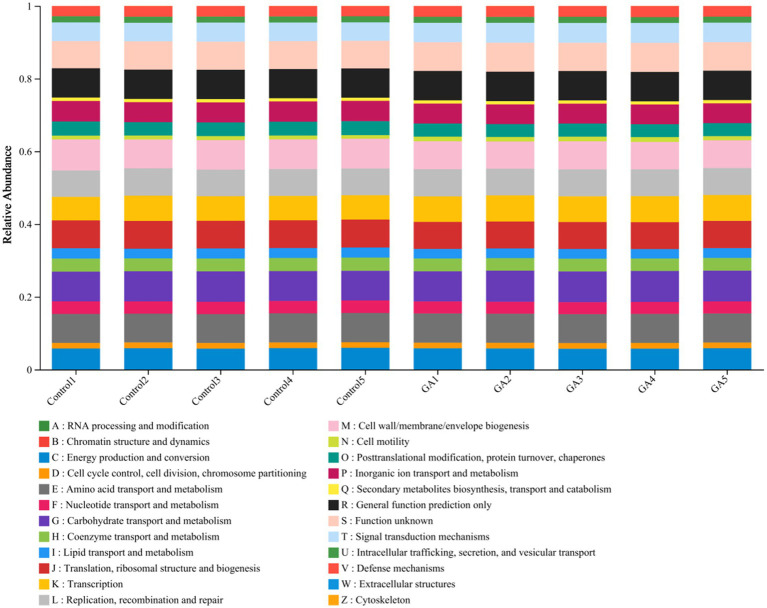
Relative abundance of PICRUSt-inferred function in GA and control treatment.

To enhance our understanding of the critical function of microbiota in two distinct groups, the PICRUSt program was used to forecast our high-throughput sequencing data based on 16S rRNA. Additionally, the analysis was conducted with reference to the Cluster of Orthologous Groups (COG) database. It is worth mentioning that the GA treatment exhibited significant enrichment in areas such as carbohydrate transport and metabolism, amino acid transport and metabolism, and energy production and conversion (*p* < 0.05; Figure 4).

## 4 Discussion

As a key production index, ADG can reflect the ability of beef cattle to digest and absorb diets. The feed/gain ratio represents the economic benefit and feed utilization efficiency, and the decrease represents the amount of feed consumed under the same weight gain condition, and the feed conversion rate is improved. In the present experiment, dietary glycyrrhetinic acid supplementation significantly increased ADG and decreased the feed-to-meat ratio in beef cattle. The findings align with those observed in sheep studies. Zhao et al. (26) indicated that incorporating glycyrrhetinic acid into the diet of sheep led to a notable rise in average daily gain (ADG) and a reduction in the feed conversion rate (FCR).

The biochemical indicators in serum indicate the physiological state of the body. Therefore, by detecting serum biochemical indicators, such as hormone levels and immune-related indicators, we can determine whether the experimental animal is in a good physiological state. In the present study, the levels of IL4 and IL6 in the GA treatment were significantly higher than in the control group. IL4, a multifunctional cytokine produced by T cells, has shown the ability to modify disease outcomes in several relevant animal model systems (27–29). Previously, we found that IL6 has a promoting effect on the treatment of various diseases (30, 31). Accordingly, combined with previous apparent nutrient digestibility results, we infer that the addition of glycyrrhetinic acid to the feed increased the concentrations of IL4 and IL6 in the blood samples, thereby improving the body’s immune response to external adverse factors, which in turn increased apparent nutrient digestibility. This result is consistent with previous studies (32, 33).

The rumen serves as a crucial digestive organ for ruminants, playing a significant role in their ability to break down complex feed materials. Within the rumen, a diverse array of microorganisms thrives, resulting in a rich microbial ecosystem that enables the fermentation of various fibrous substances. This process is essential, as many of these fibers are difficult for monogastric animals to digest effectively, highlighting the unique digestive capabilities of ruminants (34). Microorganisms in the rumen degrade protein and carbohydrates in the feed into ammonia nitrogen and volatile fatty acids, respectively. Volatile fatty acids are the hosts of the main energy source, providing more than 70% of the energy for growth and growth performance (35). In the current study, the addition of glycyrrhetinic acid to the feed significantly increased the propionate and total volatile fatty acid concentrations in the rumen and simultaneously decreased the ratio of acetate to propionate and improved the rumen fermentation pattern. In addition, the ratio of acetic acid-to-propionic acid directly affects ruminant growth performance. In dairy cows, acetic acid plays a crucial role in the production of milk fat, primarily through its absorption by the mammary gland (36), while in beef cattle, propionic acid is the main precursor of glucose synthesis (37). In this study, the proportion of propionic acid significantly increased, which was beneficial to cattle growth performance, which is consistent with ADG. From this, we can infer that the addition of glycyrrhetinic acid to the feed increases rumen volatile fatty acid content by promoting cellulose decomposition by microorganisms, thereby affecting growth performance.

In the context of this experiment, the primary phyla of rumen microorganisms are *Bacteroidetes* and *Firmicutes*, collectively comprising a relative abundance of 90%, a finding that aligns with earlier research (38).These are the main phyla that digest protein and carbohydrates in the feed for body use. Additionally, the ratios of *Bacteroidetes* to *Firmicutes* in the GA treatment were considerably lower compared to the control group. Numerous studies have demonstrated a strong correlation between the *Bacteroidota* to *Firmicutes* ratio and obesity (39), as data showed that a lower *Bacteroidota*:*Firmicutes* ratio occurred in obese but not lean mice (40). This may be related to the increased daily weight gain of the cattle in our experiment. Despite their relatively low abundance, Proteobacteria play an important role in rumen metabolism and are frequently observed in a starch-rich diet (41). *Fibrobacteria* play a crucial role in the breakdown of cellulose, and they are frequently found in diets that are high in fiber (42). In previous studies, *Proteobacteria* were usually the third most abundant (43), but in our study, their abundance decreased in favor of *Patescibacteria*. At the same time, the relative abundance of *Actinobacteriota* also increased.

At the genus level, the effects of glycyrrhetinic acid on rumen microbes were further demonstrated, with *Bacteroidetes* and *Firmicutes*, the main genera, having a relative abundance of >1%. This observation is consistent with findings from previous studies (44, 45). In this experiment, the predominant genera identified within the rumen were *Prevotella*, *Rikenellaceae_RC9_gut_treatment*, *NK4A214_group*, *Prevotellaceae_UCG-003*, *Ruminococcus*, *Lachnospiraceae_NK3A20_ group*, and some unclassified bacteria, for example, *norank_f_Bacteroidales_RF16_ group*, *norank_f_F082, norank_f_UCG-01*, and *norank_f_Euerium_coprostanoligenes_ group. Prevotella* emerged as the genus exhibiting the highest relative abundance, highlighting its significant role in the digestion of lignocellulose within the rumen (46). However, the abundance of *Prevotella* in the experimental treatment was considerably reduced compared to the control treatment, and the reason remains to be further investigated. In GA treatment, the *NK4A214_group*, *norank_f_UCG-011*, *Christensenellaceae_R-7_group*, and *Pseudobutyrivibrio* exhibited a significantly higher abundance in comparison to the control. *NK4A214_group* and *norank_f_UCG-011* were identified as having strong fiber degradation ability (47), and other scholars (48) have confirmed that elevated acetate and butyrate are associated with *Christensenellaceae_R-7_group*. *Pseudobutyrivibrio* is involved in plant fiber degradation (49). Combining the above improvements in the abundance of multiple bacteria may explain why the GA treatment had a stronger fiber-degrading ability and a higher concentration of volatile fatty acids.

Functional prediction enhances our understanding of the ecological functions of the rumen and provides valuable insights into the health status of the host (50). In the context of functional prediction, we observed significant increases in the processes of carbohydrate transport and metabolism, as well as energy production and conversion, which may explain the significant increase in the apparent digestibility of nutrients and the concentration of volatile fatty acids in the rumen fluid in the GA group in our previous experiment. Nonetheless, the underlying mechanisms require further investigation.

## 5 Conclusion

Supplementing the diet with glycyrrhetinic acid improved beef cattle growth performance, increased IL4 and IL6 serum content, increased the apparent digestibility of nutrients, and modified the rumen microbial composition and diversity of beef cattle.

## Data Availability

The datasets presented in this study can be found in online repositories. The names of the repository/repositories and accession number(s) can be found in the article/supplementary material.
